# Risk Factors for Dysphagia and the Impact on Outcome After Spontaneous Subarachnoid Hemorrhage

**DOI:** 10.1007/s12028-019-00874-6

**Published:** 2019-11-15

**Authors:** Tobias Keser, Mario Kofler, Mariella Katzmayr, Alois J. Schiefecker, Verena Rass, Bogdan A. Ianosi, Anna Lindner, Maxime Gaasch, Ronny Beer, Paul Rhomberg, Erich Schmutzhard, Bettina Pfausler, Raimund Helbok

**Affiliations:** 1 Neurological Intensive Care Unit, Department of Neurology, Medical University of Innsbruck, Anichstrasse 35, 6020 Innsbruck, Austria; 2 Medical Informatics, UMIT – University for Health Sciences, Hall in Tirol, Austria; 3 Department of Neuroradiology, Medical University of Innsbruck, Innsbruck, Austria

**Keywords:** Subarachnoid hemorrhage, Dysphagia, Outcome, Swallowing

## Abstract

**Background:**

Despite the tremendous impact of swallowing disorders on outcome following ischemic stroke, little is known about the incidence of dysphagia after subarachnoid hemorrhage (SAH) and its contribution to hospital complications, length of intensive care unit stay, and functional outcome.

**Methods:**

This is a retrospective analysis of an ongoing prospective cohort study. Swallowing ability was assessed in consecutive non-traumatic SAH patients admitted to our neurological intensive care unit using the Bogenhausen Dysphagia Score (BODS). A BODS > 2 points indicated dysphagia. Functional outcome was assessed 3 months after the SAH using the modified Rankin Scale with a score > 2 defined as poor functional outcome.

**Results:**

Two-hundred and fifty consecutive SAH patients comprising all clinical severity grades with a median age of 57 years (interquartile range 47–67) were eligible for analysis. Dysphagia was diagnosed in 86 patients (34.4%). Factors independently associated with the development of dysphagia were poor clinical grade on admission (Hunt & Hess grades 4–5), SAH-associated parenchymal hematoma, hydrocephalus, detection of an aneurysm, and prolonged mechanical ventilation (> 48 h). Dysphagia was independently associated with a higher rate of pneumonia (OR = 4.32, 95% CI = 2.35–7.93), blood stream infection (OR = 4.3, 95% CI = 2.0–9.4), longer ICU stay [14 (8–21) days versus 29.5 (23–45) days, *p* < 0.001], and poor functional outcome after 3 months (OR = 3.10, 95% CI = 1.49–6.39).

**Conclusions:**

Dysphagia is a frequent complication of non-traumatic SAH and associated with poor functional outcome, infectious complications, and prolonged stay in the intensive care unit. Early identification of high-risk patients is needed to timely stratify individual patients for dysphagia treatment.

**Electronic supplementary material:**

The online version of this article (10.1007/s12028-019-00874-6) contains supplementary material, which is available to authorized users.

## Introduction

Despite the advances in the clinical management, subarachnoid hemorrhage (SAH) is still associated with a high mortality rate and substantial morbidity [1]. Patient and disease-specific factors such as initial disease severity, hospital complications as well as the need for prolonged ventilation contribute to longer intensive care unit (ICU) stays and poor functional outcome [1–3]. Post-extubation dysphagia occurs in most patients with neurological impairment and is associated with prolonged mechanical ventilation, the development of pneumonia, and long-term morbidity [4]. Although the incidence of swallowing disorders has been extensively studied in ischemic stroke patients, little is known about the true incidence of dysphagia in SAH patients and its contribution to the clinical course and outcome.

Swallowing involves a complex sequence of neuromuscular events. Several cortical brain regions, the cerebellum and the brainstem, are involved in the processing of afferent stimuli, initiating the voluntary oral phase of swallowing and coordinating consecutive reflexive mechanisms [5–7]. All of those regions may incur functional impairment or structural damage through mechanisms of early and secondary brain injury, as well as global cerebral dysfunction following SAH. Furthermore, brainstem dysfunction without obvious underlying brain pathology has been described in SAH patients [8].

So far, the incidence of dysphagia in SAH patients has been reported to range between 31.6 and 46.8% [9, 10]. Several risk factors for the development of dysphagia were identified; however, the impact of impaired swallowing on functional outcome remains poorly described.

In ischemic stroke patients, dysphagia is one of the most important determinants of functional outcome and self-sufficient living. In SAH, the early identification of risk factors for the development of dysphagia may help to timely allocate resources to individual patients. Furthermore, the novel understanding of neuronal plasticity of brain regions involved in the swallowing process has led to new treatment approaches, such as electrical pharyngeal stimulation, which can be applied already early after ictus. This intervention was recently found to be associated with an improved decannulation rate in tracheotomized stroke patients [11]. In addition, oropharyngeal air-pulse application was associated with increased resting swallowing rates in tube-fed patients with hemispheric stroke [12]. Such treatments could potentially be applied in mechanically ventilated patients as an attempt to early initiate dysphagia therapy in high-risk patients.

The main goal of the current study was (1) to quantify the rate of swallowing disorders after SAH by using a simple clinical assessment tool, (2) to identify early predictors of dysphagia in all severity grades of SAH patients, and (3) to evaluate how dysphagia contributes to hospital complications, length of ICU stay, and poor outcome. We hypothesized that dysphagia would be independently associated with higher rates of infectious complications, a longer stay in the ICU, and poor functional outcome.

## Materials and Methods

### Patient Population and Critical Care Management

Two-hundred and seventy consecutive patients with non-traumatic SAH admitted to the neurological ICU of our tertiary referral center (Medical University of Innsbruck, Austria) between 2010 and 2016 were screened. The conduct of this study was approved by the local ethics committee (Medical University Innsbruck, AN3898 285/4.8, AM4091-292/4.6), and informed consent was obtained from all patients according to federal regulations.

Twenty patients were excluded due to a lack of information on swallowing ability, because of early death or repatriation, leaving 250 patients for the final analysis. Standard critical care conformed to current international guidelines [13, 14], with the exception of intravenous instead of oral nimodipine application in poor-grade SAH patients. Ruptured aneurysms were secured by neurosurgical clipping or endovascular coiling. Transcranial color-coded duplex sonography was routinely performed for the detection of vasospasm. Catheter cerebral angiography was performed in patients with severe sonographic vasospasm (> 180–200 cm/s mean flow velocity or a Lindegaard ratio > 6), and intraarterial nimodipine treatment was considered. Patients who developed severe vasospasm or delayed cerebral ischemia were treated with blood pressure augmentation, ventilator settings to achieve a pCO_2_ > 40 mmHg and normothermia.

### Grading and Definitions

Clinical disease severity was graded using the Hunt & Hess (H&H) scale [15]. H&H grades 1–3 were considered as good grade and H&H grades 4–5 as poor-grade patients. Cerebral computed tomography (CT) scans were analyzed by a neuroradiologist using the modified Fisher scale and screening it for the presence of parenchymal hemorrhage [16]. Delayed cerebral ischemia was defined as the occurrence of a new focal neurological deficit, a decrease of ≥ 2 points on the Glasgow Coma Scale or a new infarct on CT or magnetic resonance imaging (MRI) scans not attributable to other causes [17]. Infectious complications were diagnosed using the Centers for Disease Control and Prevention criteria. The variable *prolonged intubation* was defined as duration of mechanical ventilation of more than 48 h in order to exclude patients with short-time intubation for aneurysm treatment. Functional outcome was prospectively evaluated by a study nurse, blinded to the clinical course of patients, 3 months after SAH using the modified Rankin Scale (mRS) score, and categorized into good (mRS score 0–2) and poor (mRS score 3–6) functional outcome.

### Dysphagia

Swallowing ability was routinely assessed by our speech therapists and quantified using the *Bogenhausen Dysphagia Score* (BODS), a score grading the ability to swallow saliva (BODS-1) and the possibility of oral food intake (BODS-2). The overall score (BODS-1 + BODS-2) ranges from 2 (no dysphagia) to 16 (most severe dysphagia). The full BODS description is available in Table e-1. A BODS ≥ 3 indicates dysphagia [18]. We considered a patient as dysphagic, if at least one BODS ≥ 3 was documented during neuro-ICU stay. Furthermore, dysphagia was subclassified using the worst documented BODS during the ICU stay as mild to moderate (BODS 3–9) or severe (BODS 10–16), as proposed by the German Society for Neurology (https://www.dgn.org/images/red_leitlinien/LL_2008/archiv/ll08kap_098.pdf). A total BODS of 10 or greater implies that patients either (1) require predominantly artificial (enteral or parenteral) nutrition, (2) have a tracheal cannula, or (3) both. There was no systematic BODS evaluation at 3 months follow-up.

### Data Collection and Analysis

Baseline characteristics, admission variables, interventions, hospital complications, and measures of functional outcome were prospectively collected in our institutional SAH database. Data on swallowing ability were obtained retrospectively from our electronic patient chart, where documentation of dysphagia using the BODS is performed by our speech therapists as a part of clinical routine patient management.

Continuous variables are reported as median and interquartile range (IQR). Categorical variables are reported as counts and proportions (%) in each group. Univariate comparisons between patients with and without dysphagia were performed using the Chi-square test for categorical variables and the Wilcoxon rank-sum test for continuous variables, as they were not normally distributed. In a second step, factors associated with dysphagia in univariate analysis (*p* < 0.05) were included in a stepwise backward elimination binary logistic regression model with the dichotomized dysphagia variable as dependent variable to identify factors independently associated with the development of dysphagia. In the same way, factors associated with poor functional outcome were identified in univariate analyses (Chi-square test, Wilcoxon rank-sum test, as appropriate) and included in a stepwise backward elimination binary logistic regression model with the dichotomized outcome variable as dependent variable. The association between dysphagia and length of ICU stay was assessed in a linear logistic regression model. All analyses were performed with IBM-SPSS (IBM SPSS Statistics, Version 24.0. Armonk, NY, USA). A *p* value smaller than 0.05 was considered as statistically significant.

## Results

Baseline characteristics, admission variables, hospital complications, and outcomes of 250 patients with non-traumatic SAH are shown in Table 1. Dysphagia was diagnosed in 86 patients (34.4%) of which 62 (72%) had severe dysphagia. Overall, the diagnosis was made after a median of 15.5 (IQR 9–24) days and, in patients who were intubated, 1 (IQR 1–4) day after extubation. Percutaneous endoscopic gastrostomy (PEG) was placed in 44 patients (17.6%), of whom two had mild or moderate and 42 had severe dysphagia, corresponding to a PEG rate of 8.3% (2/24 patients with mild or moderate dysphagia) and 67.7% (42/62 patients with severe dysphagia). Forty patients (23.4%) admitted with good clinical grade and 46 patients (58.2%) with poor initial clinical grade developed dysphagia. In 120/250 patients (48%), no focal brain lesion was detected on any CT or MRI scan during the ICU stay. Of those, 21 (18%) suffered from dysphagia.

**Table 1:** Baseline characteristics, hospital complications, and outcome

Patient characteristics	*n* (%) or median (IQR)			*p* value (no dysphagia vs. dysphagia)	*n* (%) or median (IQR)		*p* value (mild/moderate vs. severe dysphagia)
	All (*n* = 250)	No Dysphagia (*n* = 164)	Dysphagia (*n* = 86)		Mild/moderate dysphagia (*n* = 24)	Severe dysphagia (*n* = 62)	
Age, years, median (IQR)	57 (47–67)	54.5 (46-63)	61 (52-71)	0.003*	63 (55-71.5)	61 (50-70.5)	0.855*
Female sex, *n* (%)	156 (62)	94 (57)	62 (72)	**0.022**^**†**^	18 (75)	44 (71)	0.708†
Hunt & Hess grade on admission, *n* (%)				**<0.001**‡			**<0.001**‡
1	72 (29)	62 (38)	10 (12)		6 (25)	4 (7)	
2	52 (21)	43 (26)	9 (11)		4 (17)	5 (8)	
3	47 (19)	26 (16)	21 (24)		9 (37)	12 (19)	
4	16 (6)	8 (5)	8 (9)		0 (0)	8 (13)	
5	63 (25)	25 (15)	38 (44)		5 (21)	33 (53)	
Aneurysm detected, *n* (%)	192 (77)	111 (77)	81 (94)	**<0.001**^**†**^	23 (96)	58 (94)	0.685^†^
Aneurysm location, *n* (%)				0.875^†^			0.566^†^
Anterior circulation	139 (72)	80 (73)	59 (72)		17 (74)	42 (71)	
Posterior circulation	53 (28)	30 (27)	23 (28)		6 (26)	17 (29)	
Aneurysm treatment				0.467^†^			0.584^†^
Endovascular coiling, *n* (%)	124 (50)	76 (46)	49 (57)		15 (63)	34 (55)	
Neurosurgical clipping, *n* (%)	65 (26)	33 (20)	32 (37)		8 (33)	24 (39)	
Modified Fisher score on admission, *n* (%)				**<0.001**‡			0.063‡
1	33 (13)	30 (18)	3 (3.5)		1 (4)	2 (3)	
2	37 (15)	26 (16)	11 (13)		3 (12.5)	8 (13)	
3	62 (25)	46 (28)	16 (18.5)		5 (21)	11 (18)	
4	118 (47)	62 (38)	56 (65)		15 (62.5)	41 (66)	
Parenchymal hemorrhage on admission CT, *n* (%)	44 (18)	17 (10)	27 (31)	**<0.001**^**†**^	7 (29)	20 (32)	0.747^†^
Delayed cerebral ischemia, *n* (%)	47 (19)	24 (15)	23 (27)	**0.020**^**†**^	3 (13)	20 (32)	0.063^†^
Hydrocephalus requiring EVD placement, *n* (%)	120 (48)	58 (35)	62 (72)	**<0.001**^**†**^	17 (71)	45 (73)	0.871^†^
Prolonged intubation, *n* (%)	140 (56)	59 (36)	81 (94)	**<0.001**^**†**^	20 (83)	61 (98)	**<0.007**^**†**^
Pneumonia, *n* (%)	109 (44)	48 (29)	61 (71)	**<0.001**^**†**^	15 (63)	46 (74)	0.284^†^
Blood stream infection, *n* (%)	40 (16)	13 (8)	27 (31)	**<0.001**^**†**^	4 (17)	23 (37)	0.067^†^
Ventriculitis, *n* (%)	31 (12)	16 (10)	15 (17)	0.080^†^	4 (17)	11 (18)	0.906^†^
Tracheotomy, *n* (%)	45 (18)	0 (0)	45 (52)	**<0.001**^**†**^	1 (4)	44 (71)	**<0.001**^**†**^
Length of mechanical ventilation, days, median (IQR)	4 (0–13)	1 (0–7)	15.5 (7–24)	<0.001*	5.5 (4–13)	20 (11–27)	<0.001*
Length of ICU stay, days, median (IQR)	18 (11–29)	14 (8–21)	30 (23–45)	<0.001*	22 (16–28)	37.5 (26–49)	<0.001*
Modified Rankin Scale score after 3 months, *n* (%)				**<0.001**‡			**<0.001**‡
0	59 (24)	55 (33.5)	4 (5)		4 (17)	0 (0)	
1	59 (22)	51 (31)	8 (9)		4 (17)	4 (6)	
2	34 (14)	19 (12)	15 (17)		8 (33)	7 (11)	
3	21 (9)	10 (6)	11 (13)		4 (17)	7 (11)	
4	19 (8)	9 (5.5)	10 (12)		3 (12)	7 (11)	
5	38 (15)	13 (8)	25 (29)		1 (4)	24 (40)	
6	20 (8)	7 (4)	13 (15)		0 (0)	13 (21)	

Prolonged intubation was defined as intubation for more than 48 h

Bold *p*-values indicate significant associations (*p* < 0.05)

*CT* computed tomography, *EVD* external ventricular drain, *ICU* intensive care unit, *IQR* interquartile range

Statistical analysis was performed using the Wilcoxon rank-sum test (*), the Chi-square test (†), and the linear-by-linear association test for trend (‡)

### Factors associated with the development of dysphagia

Factors associated with dysphagia in univariate analysis are shown in Table 1. The risk of developing dysphagia did not differ between aneurysm securing methods (*p* = 0.567) or aneurysm locations (*p* = 0.875). Based on the results of the univariate analysis, age, sex, Hunt & Hess grade, detection of an aneurysm, modified Fisher score, parenchymal hemorrhage, delayed cerebral ischemia, hydrocephalus, prolonged intubation, pneumonia, and blood stream infection were the variables included in the multivariable analysis assessing independent associations with the development of dysphagia. Those results are presented in Table 2.

**Table 2:** Factors associated with the development of dysphagia in multivariable analysis

Factor	OR	95% CI	*p* value
Poor clinical grade	2.02	1.03–3.96	0.040
Parenchymal hematoma on admission CT	2.55	1.17–5.53	0.018
Hydrocephalus requiring EVD placement	2.60	1.33–5.10	0.005
Aneurysm detected	3.48	1.22–9.92	0.019
Prolonged intubation	19.2	6.18–59.8	<0.001

Poor clinical grade was defined as Hunt & Hess grades 4 and 5. Prolonged intubation was defined as intubation for more than 48 h. Statistical analysis was performed using a stepwise backward elimination binary logistic regression model

*CI* confidence interval, *CT* computed tomography, *EVD* external ventricular drain, *OR* odds ratio

### Dysphagia and Outcome

**Fig. 1:**
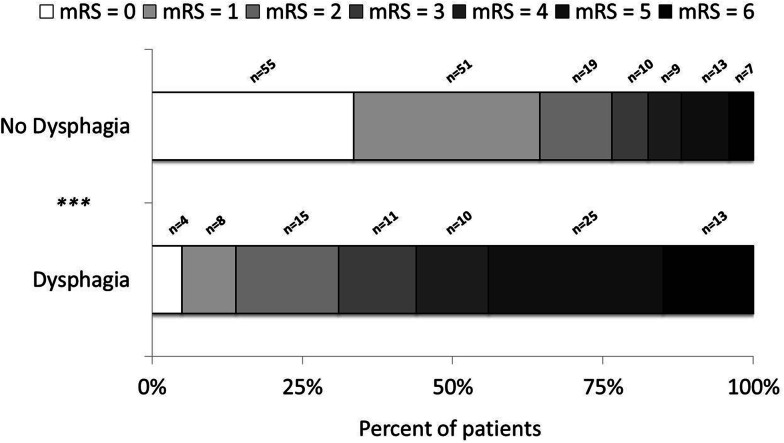
Functional outcome after 3 months (assessed using the modified Rankin Scale) in patients with and without dysphagia during the intensive care unit stay. The asterisks (***) indicate a significant difference (*p* < 0.001) in outcomes between groups (linear-by-linear association test); mRS = modified Rankin Scale

Ninety-eight patients (39%) had a poor functional outcome after 3 months. We found a significant association between dysphagia and poor functional outcome (univariate OR 7.0, 95% CI = 3.9–12.5, *p* < 0.001). The detailed distribution of functional outcomes in patients with and without dysphagia is shown in Fig. 1. Furthermore, we identified dysphagia as being independently associated with poor functional outcome (Table 3).

**Table 3:** Factors independently associated with poor functional outcome after SAH

Factor	OR	95% CI	*p* value
Pneumonia	2.85	1.40–5.80	0.004
Dysphagia	3.10	1.49–6.39	0.002
Poor clinical grade	3.48	1.66–7.31	0.001
Age above median	5.21	2.50–10.9	< 0.001

Poor clinical grade was defined as Hunt & Hess grades 4 and 5. Median age was 57 years. Statistical analysis was performed using a stepwise backward elimination binary logistic regression model

*CI* confidence interval, *OR* odds ratio

In addition, patients with severe dysphagia had a higher risk of poor functional outcome (adj. OR = 6.87, 95% CI = 2.17–21.7, *p* < 0.001) when compared to SAH patients with mild/moderate dysphagia. Moreover, dysphagia was associated with a higher rate of pneumonia (adj. OR = 4.32, 95% CI = 2.35–7.93, *p* < 0.001), blood stream infection (adj. OR = 4.3, 95% CI = 2.0–9.4, *p* < 0.001), and a longer ICU stay (14 [8–21] days versus 29.5 [23–45] days, *p* < 0.001). The statistical models with severe dysphagia, pneumonia, blood stream infection, and length of stay were adjusted for H&H grade and age.

Within the group of dysphagic patients, PEG placement (as an indicator of persistent dysphagia) was associated with poor functional outcome (OR = 5.41, CI 95% = 1.64–17.86, *p* = 0.006), adjusted for H&H grade and age. When patients with PEG placement were excluded from the analysis, dysphagia was still associated with poor outcome in the overall cohort (OR = 2.90, CI 95% = 1.24–6.78, *p* = 0.014).

Patients with poor outcome after 3 months had a significantly higher median worst BODS compared to patients with good outcome (4 [IQR 2–12] versus 2 [IQR 2–2], *p* < 0.001). There was an association between a higher BODS and poor functional outcome (OR per BODS point = 1.21, 95% CI = 1.12–1.32, *p* < 0.001), adjusted for H&H grade and age.

## Discussion

In this study, we investigated the frequency of dysphagia in all clinical severity grades of SAH patients using a simple score for the assessment of swallowing disorders in critical care patients. The main findings were that (1) dysphagia was diagnosed in every third SAH patient, including patients admitted in good clinical grade, that (2) dysphagia was associated with radiographic and clinical disease severity on admission, and (3) that dysphagia was strongly associated with hospital complications, prolonged ICU stay, and poor functional outcome.

Although it is well known that the high incidence of swallowing disorders after ischemic stroke contributes to impaired quality of life and poor functional outcome, only few studies report the incidence of dysphagia following SAH [9, 10]. In a retrospective cohort study including predominantly good-grade SAH patients, the incidence was 31.6%, which is comparable to our findings [9]. It is important to mention that even patients presenting with good clinical grades on admission are at risk of developing swallowing disorders, especially when prolonged mechanical ventilation is needed. Our data may help to identify patients at high risk early in order to allocate specific resources already early after extubation or even during the time of mechanical ventilation.

Known risk factors for the development of dysphagia after SAH include older age, initial disease severity, the amount of cisternal and intraventricular blood, detection of an aneurysm, rebleeding, hydrocephalus, vasospasm, cerebral ischemia, and mechanical ventilation [9, 10]. We additionally identified parenchymal hematoma as being independently associated with the development of dysphagia. However, our data and previous studies indicate that swallowing disorders after SAH are not only due to structural brain damage, but also occur in patients without focal parenchymal injury (18% in our cohort), possibly owing to global cerebral dysfunction after SAH and/or local mechanical irritation during intubation. In our cohort, intubation for more than 48 h was by far the strongest predictor of dysphagia, which certainly needs a differentiated interpretation. Dysphagia after intubation is a well-known complication with an incidence exceeding 20% in most studies, even in non-neurological patients [19]. One mechanism seems to be mechanical irritation of the pharynx and larynx, as, among others, prolonged intubation, increased operative time, multiple intubations, and perioperative transesophageal echocardiography have been identified as risk factors for dysphagia after intubation [19]. Notably, patients suffering from severe SAH with poor admission clinical grades, and those with parenchymal hematoma, hydrocephalus, or detection of an aneurysm (data not shown) more frequently required prolonged intubation. Therefore, despite being of independent statistical significance, prolonged intubation may simply reflect disease severity in our cohort.

Another entity associated with a higher incidence of dysphagia is critical illness polyneuropathy and myopathy [20]. Unfortunately, we only performed electroneurographic studies in poor-grade patients with high clinical suspicion, which made it impossible to further elucidate this association.

Delayed cerebral ischemia was a univariate predictor of swallowing disturbance, but lost its significance in multivariable analysis. This may be due to small ischemic areas and infarction outside of brain regions involved in the swallowing process.

Here, we established a strong association between dysphagia and poor functional outcome after 3 months. This is in line with findings in ischemic stroke patients, in whom dysphagia was associated with an increased risk of death, a higher rate of disability, longer hospitalization, and consecutive need for institutional care [21, 22]. Moreover, this study demonstrates that there is a strong association between dysphagia severity and the probability of poor functional outcome, reflected by the association of a higher BODS and PEG placement with poor outcome. Importantly, dysphagia was still associated with poor outcome in the overall cohort when patients with PEG placement (an indicator of persistent dysphagia) were excluded from the analysis. This signifies that also transient dysphagia negatively impacts on functional outcome. In this cohort, dysphagia and pneumonia were the only modifiable risk factors contributing to poor functional outcome in the logistic regression model. Our findings underline the importance of early diagnosis and treatment of dysphagia in SAH patients.

Conventional treatment options for dysphagic patients include texture-modified diets, speech and language therapy programs, non-oral (enteral) feeding, medication, as well as physical and olfactory stimulation [23]. Recent studies demonstrated experience-dependent neuronal plasticity of brain regions involved in the process of swallowing [24], which led to the development of several experimental interventions such as stimulation of the pharynx with air pulses. Increased resting swallowing rates in tube-fed stroke patients with dysphagia have been shown in these patients [12]. Moreover, pharyngeal electrical stimulation favorably influenced the decannulation rate in tracheotomized ischemic stroke patients [11]. These novel approaches may be of special interest in SAH patients, as prolonged mechanical ventilation is common in poor admission grade patients rendering an early time window for these interventions.

Some limitations merit consideration. Although every patient was evaluated for swallowing disorders, the time from ictus to assessment largely differed between patients as the BODS assessment requires a certain level of vigilance and cooperation, as well as a clinically stable condition, which also explains the comparably long time to diagnosis. Furthermore, assessment of swallowing disorders was not available on weekends, which may have led to a delay in diagnosis in individual patients. We only included the worst BODS in the final analysis as, due to the retrospective nature of the study, consecutive BODS data of sufficient quality were not available in all patients. On the other hand, this approach may illustrate the negative impact on outcome even of temporary dysphagia. Importantly, the BODS was not assessed in patients with metabolic encephalopathy or delirium. In summary, prospective data with predefined intervals of BODS assessment are needed to further elucidate the association between dysphagia and functional outcome in SAH. Furthermore, we used fiber-endoscopic evaluation of swallowing only in patients with severe swallowing disorders, which diminishes the comparability of our results with studies assessing dysphagia with instrumental methods. The strength of the BODS evaluation is its simplicity, and it can be applied in all hospitals independent of available equipment.

## Conclusion

Dysphagia is a frequent complication of non-traumatic subarachnoid hemorrhage and it is associated with poor functional outcome, infectious complications, and prolonged stay at the ICU. Early identification of high-risk patients may be possible and opens the opportunity for early treatment, maybe even in unconscious and intubated patients. Further investigations are needed to prove the efficacy of such interventions. This study argues for comprehensive dysphagia screening in all clinical severity grades of SAH patients.

## Electronic supplementary material

Below is the link to the electronic supplementary material.

Supplementary material 1 (DOC 49 kb)
