# Supplementary material for: Risk Factors for Dysphagia and the Impact on Outcome After Spontaneous Subarachnoid Hemorrhage
Source: Neurocrit Care. 2019 Nov 15;33(1):132–9. doi: 10.1007/s12028-019-00874-6 (PMC7392368; doi:10.1007/s12028-019-00874-6)
Supplement: Supplementary file 1 — Supplementary material 1 (DOC 49 kb) [file 12028_2019_874_MOESM1_ESM.doc]

**Table e-1 (Supplemental Table 1) - Bogenhausen dysphagia score (BODS)**

| **BODS-1** | **Impairment of swallowing saliva** |
| --- | --- |
|  |  |
| 1 | No tracheal cannula, efficient swallowing of saliva (normal). |
| 2 | No tracheal cannula, inefficient swallowing of saliva, occasionally gurgling voice and/or expectorations (less than 1 / hour), sufficient protective mechanisms. |
| 3 | No tracheal cannula, inefficient swallowing of saliva, frequently gurgling voice and/or expectorations (more than 1 / hour), sufficient protective mechanisms. |
| 4 | No tracheal cannula, insufficient protective mechansims, occasional suctioning necessary **OR** permanently unblocked cannula **OR** tracheostomy placeholder. |
| 5 | Tracheal cannula, unblocked for more than 12 but less than 24 hours / day. |
| 6 | Tracheal cannula, unblocked for more than 1 but less than 12 hours / day. |
| 7 | Tracheal cannula, unblocked for 1 hour / day or less. |
| 8 | Tracheal cannula, permanently blocked. |
|  |  |
| **BODS-2** | **Impairment of oral food intake** |
|  |  |
| 1 | Full oral nutrition without limitations (normal). |
| 2 | Full oral nutrition with minor limitations: several consitencies without compensatory techniques **OR** compensatory techniques without dietary limitations. |
| 3 | Full oral nutrition with moderate limitations: several consitencies and compensatory techniques. |
| 4 | Full oral nutrition with grave limitations: only 1 consistency with or without compensatory techniques. |
| 5 | Predominantly oral nutrition (1 meal or more / day), supplemental enteral or parenteral nutrition. |
| 6 | Partial oral nutrition (more than 10 spoons but less than 1 meal / day), predominantly enteral or parenteral nutrition. |
| 7 | Minimal oral nutrition (less than 10 spoons / day), predominantly enteral or parenteral nutrition. |
| 8 | Exclusively enteral or parenteral nutrition. |
